# Ultrasound images-based deep learning radiomics nomogram for preoperative prediction of *RET* rearrangement in papillary thyroid carcinoma

**DOI:** 10.3389/fendo.2022.1062571

**Published:** 2022-12-20

**Authors:** Jialong Yu, Yihan Zhang, Jian Zheng, Meng Jia, Xiubo Lu

**Affiliations:** ^1^ Department of Thyroid Surgery, The First Affiliated Hospital of Zhengzhou University, Henan, China; ^2^ Department of Ophthalmology, The First Affiliated Hospital of Zhengzhou University, Henan, China

**Keywords:** papillary thyroid carcinoma, radiomics, deep learning, nomogram, *RET* rearrangement, prediction

## Abstract

**Purpose:**

To create an ultrasound -based deep learning radiomics nomogram (DLRN) for preoperatively predicting the presence of *RET* rearrangement among patients with papillary thyroid carcinoma (PTC).

**Methods:**

We retrospectively enrolled 650 patients with PTC. Patients were divided into the *RET*/PTC rearrangement group (n = 103) and the non-*RET*/PTC rearrangement group (n = 547). Radiomics features were extracted based on hand-crafted features from the ultrasound images, and deep learning networks were used to extract deep transfer learning features. The least absolute shrinkage and selection operator regression was applied to select the features of nonzero coefficients from radiomics and deep transfer learning features; then, we established the deep learning radiomics signature. DLRN was constructed using a logistic regression algorithm by combining clinical and deep learning radiomics signatures. The prediction performance was evaluated using the receiver operating characteristic curve, calibration curve, and decision curve analysis.

**Results:**

Comparing the effectiveness of the models by linking the area under the receiver operating characteristic curve of each model, we found that the area under the curve of DLRN could reach 0.9545 (95% confidence interval: 0.9133–0.9558) in the test cohort and 0.9396 (95% confidence interval: 0.9185–0.9607) in the training cohort, indicating that the model has an excellent performance in predicting *RET* rearrangement in PTC. The decision curve analysis demonstrated that the combined model was clinically useful.

**Conclusion:**

The novel ultrasonic-based DLRN has an important clinical value for predicting *RET* rearrangement in PTC. It can provide physicians with a preoperative non-invasive primary screening method for *RET* rearrangement diagnosis, thus facilitating targeted patients with purposeful molecular sequencing to avoid unnecessary medical investment and improve treatment outcomes.

## 1 Introduction

Thyroid cancer is the most common endocrine tumor; papillary thyroid carcinoma (PTC) is the most common type of pathological cancer, accounting for approximately 80%–90% of all thyroid cancers (1). Yasuhiro et al. studied 5897 patients with PTC and reported that PTC is inert cancer with a low mortality rate and >90% overall survival rate (2). However, some histological subtypes of PTC show aggressive behavior, have a high recurrence and distant metastasis, or lead to death (3, 4). Therefore, early discrimination against these PTCs that require aggressive medical intervention is important.

Several genetic alterations have been used as a tool for diagnosing diseases and predicting prognosis owing to the advancement in molecular genetics (5, 6). On a molecular basis, some genetic alterations are closely associated with the clinicopathological features of PTC. Fusco et al. first reported the *RET* chromosomal rearrangement was in PTC. *RET* is a proto-oncogene that encodes a plasma membrane-bound *RET* tyrosine kinase receptor for ligands of the glial-derived neurotrophic factor family (7). Chromosomal rearrangements cause *RET*/PTC-related carcinogenesis (8).

Thus far, at least 13 different forms of *RET*/PTC rearrangements have been found; these rearrangements are almost exclusively found in PTC (9). Among all rearrangement forms, *RET*/PTC3 and *RET*/PTC1 are the most common, accounting for >90% of all rearrangements. The prognostic role of *RET* rearrangements has been confirmed in other studies as the presence of *RET*/PTC3 rearrangements and both large tumors size and advanced tumor stage at the time of diagnosis are positively associated; these studies highlight that *RET*/PTC3 has a significant role in metastatic spread (10–13). However, *RET*/PTC1 rearrangement is more prevalent than *RET*/PTC3 in less aggressive classical variants (14, 15).

Ultrasound is the primary imaging technique for the evaluation of thyroid nodules. Predicting molecular alterations in PTC by analyzing conventional ultrasound features is controversial, mainly due to the limitation of conventional ultrasound images and high interobserver variability (16). Radiomics can automatically extract innumerable high-dimensional features from images; however, these features cannot be assessed visually. Radiomics has recently shown clinical importance in the thyroid (17). Radiomics based on ultrasound images has been used to predict molecular properties in thyroid cancer (18–20).

Machine learning is the scientific technique that emphasizes how computers learn from data. It can be found at the intersection of statistics and computer science (21). Deep transfer learning (DTL) is a new type of machine learning method developed *via* the advancement of artificial neural networks. DTL depends on a network of computational units that gradually extract higher-lever features from the input data (22). DTL is widely used in medicine, including in the field of the thyroid (23, 24).

To our knowledge, there are no published studies aimed at identifying the presence of *RET* rearrangement in PTC using ultrasound radiomics combined with DTL. Therefore, we evaluated the association between *RET* rearrangement and ultrasound radiomics DTL and established a deep learning radiomics nomogram (DLRN) to predict *RET* rearrangement in PTC.

## 2 Materials and methods

### 2.1 Ethics statement

This study was approved by the Ethics Committee of the First Affiliated Hospital of Zhengzhou University (Number: 2022-KY-1002-002).

### 2.2 Clinical data

**Figure 1:**
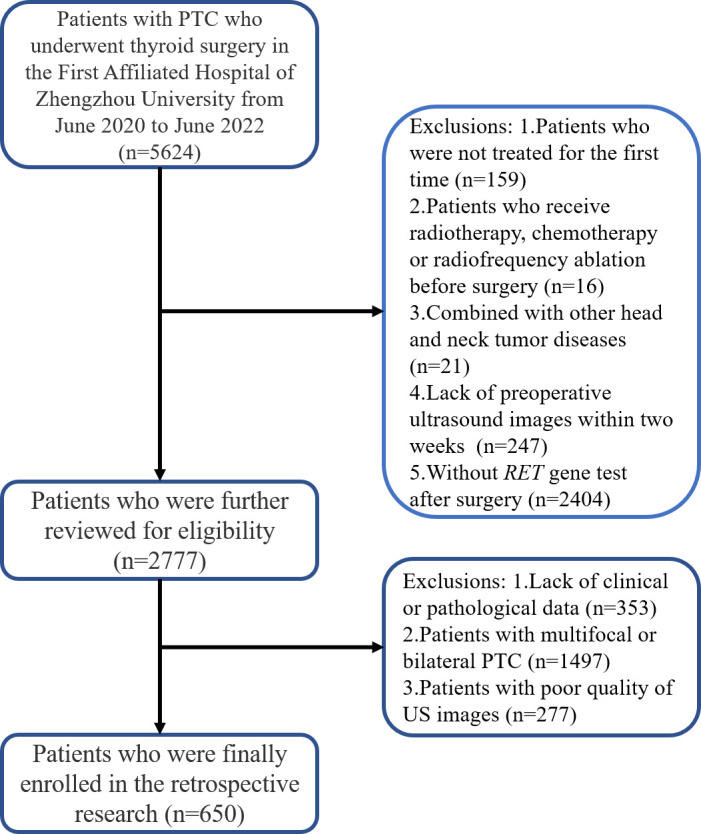
The patient recruitment pathway.

Between June 2020 and June 2022, we enrolled consecutive patients from the Department of Thyroid Surgery of the First Affiliated Hospital of Zhengzhou University; informed consent from patients was exempted. The inclusion criteria were as follows: (1) patients who were treated for the first time; (2) patients who preoperatively underwent ultrasound examination within 2 weeks; (3) patients who had pathologically confirmed PTC; and (4) patients with well-preserved clinical data, imaging data, and pathological specimens. The exclusion criteria were as follows: (1) patients who underwent preoperative radiotherapy, chemotherapy, or radiofrequency ablation; (2) patients who presented with other head and neck tumor diseases; (3) patients with multifocal or bilateral PTC; and (4) patients with poor quality ultrasound images. **Figure 1** shows the patient recruitment pathway. The patients were divided into training and test cohorts using a 5-fold cross-validation method.

### 2.3 Ultrasound examination and image acquisition

All enrolled patients underwent a preoperative neck ultrasound examination. The ultrasound machines included HITACHI HI VISION Ascendus (Japan), TOSHIBA aplio500 (Japan), SAMSUNG LA3-16A (Korea), and PHILIPS RPIQ5 (Netherlands). The ultrasound examinations were performed with a 5–12 MHz transducer by radiologists with 5–10 years of experience in thyroid ultrasound evaluation. After placing the patients in the supine position, longitudinal and transverse continuous scanning were performed to obtain longitudinal and transverse images of the thyroid nodules. All selected thyroid nodules were evaluated for the following ultrasound feature composition (mixed cystic and solid, cystic, or solid), echogenicity (hypoechoic, isoechoic, or hyperechoic), tumor margin (irregular, ill-defined, or smooth), vertical and horizontal diameter ratio (<1 or ≥1), shape (irregular, ill-defined, or regular), and calcification (macrocalcification, microcalcification, non-calcification, or cluster calcification); the American College of Radiology Thyroid Imaging Reporting and Data System (ACR-TI-RADS) score of each nodule was calculated by the same radiologists. **Supplement Tables 1**, **2** show the detailed process of calculating the ACR-TI-RADS score and category.

### 2.4 Region of interest segmentation

Two radiologists with >3 years of experience who were blinded to the pathological results reviewed the ultrasound images of the enrolled patients using Picture Archiving and Communication Systems, selected appropriate images, stored the selected images in BMP format, and then converted them to NII format. The open-source software 3D Slicer (version 4.1.13.0, available at https://www.slicer.org/) was used for texture analysis. The region of interest of the target nodule was manually segmented using a 3D Slicer. The interobserver and intraobserver agreements were measured using random 130 nodules delineated by a radiologist twice within 2 weeks. The interclass correlation coefficient was used to evaluate the interobserver and intraobserver agreement of the feature extraction. An interclass correlation coefficient larger than 0.75 was considered a satisfactory agreement.

### 2.5 Features extraction and signature building

All handcrafted features were extracted using an in-house feature analysis program implemented in Pyradiomics (http://pyradiomics.readthedocs.io); 1477 handcrafted features were extracted from each ultrasound image. After the least absolute shrinkage and selection operator (LASSO) feature screening, we input the final features into the machine learning models such as LR, SVM, random forest, XGBoost, and so on for prediction model construction.

Deep learning features were extracted from pre-trained convolutional neural networks *via* transfer learning. In this study, resnet50 was chosen as the pre-trained convolutional neural network model18. The resnet50 model was trained on the ILSVRC-2012 dataset. The image that had the largest tumor area was selected to represent each patient; the gray values were normalized to the range [−1, 1] using min-max transformation. Then, each cropped subregion image was resized to 224 × 224 with the nearest interpolation. The obtained images were used as the model input. Since the dimension of deep migration features was 2048, we used the principal component analysis to reduce the dimension of deep migration features and ensure the balance between features. We reduced the dimension of deep learning to 128 dimensions for improving the generalization ability of the model and reducing the risk of overfitting. After compressing the deep learning feature by principal component analysis, all compressed features were standardized using the Z-score method, and the mean and variance (standard deviation) of each column of features were calculated. Each column of features was subtracted from the mean, divided by variance, and transformed into a standard normal distribution. We used the least absolute shrinkage and selection operator (LASSO) to filter out features whose coefficients were not 0, selected and reduced the dimension of fusion features, and obtained the optimal subset of fusion features.

Based on the selected radiomics features and 128 compressed DTL features, we aimed to create a deep learning radiomics (DLR) signature. We followed the same path as the radiomics signature or DTL signature. After LASSO feature screening, we input the final features into the machine learning models for predictive model construction to obtain the final DLR signature.

### 2.6 Construction of DLRN and predictive performance

We referred to clinical data with the conventional ultrasound features commonly referred to as clinical features. First, the features used for building clinical signatures were selected by baseline statistics with a *p*-value of <0.05. We also used the same machine learning model in the radiomics signature-building process.

**Figure 2:**
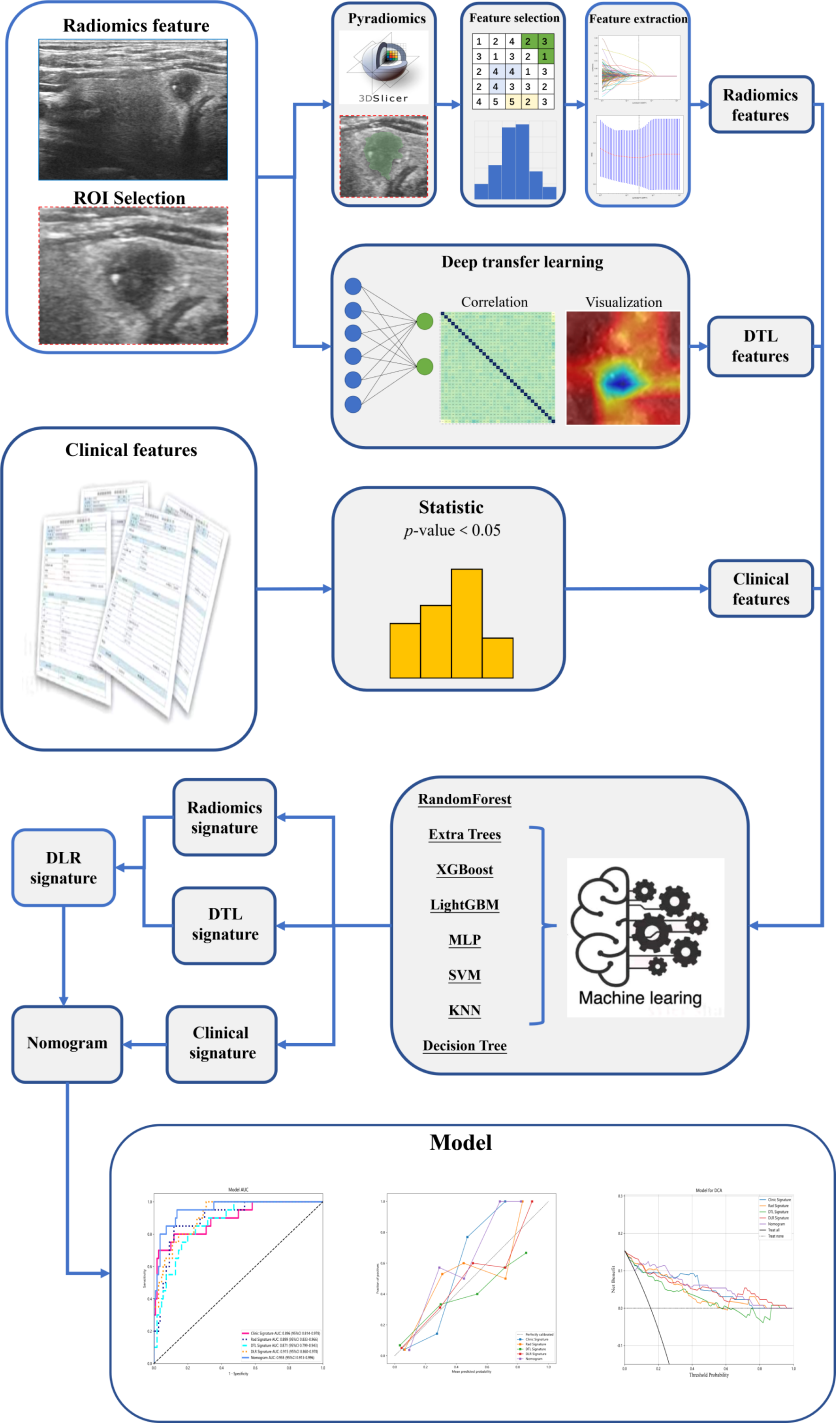
The process of model building.

DLRN was prepared in combination with clinical and DLR signatures. The diagnostic efficacy of the nomogram was tested in the test cohort; the receiver operating characteristic curves were drawn to assess the diagnostic efficacy of the nomogram. The calibration efficiency of the nomogram was estimated by drawing calibration curves; the Hosmer–Lemeshow analytical fit was used to evaluate the calibration ability of the nomogram. Decision curve analysis (DCA) was mapped to evaluate the clinical utility of predictive models. **Figure 2** shows the whole process of model building.

### 2.7 Detection of *RET*/PTC rearrangements

Genomic DNA was extracted from postoperative specimens using AmoyDX provided by Amoy Diagnostics Co., Ltd. (Xiamen, China). *RET* rearrangements were analyzed using the next-generation sequencing method. Amplification and analysis were conducted on an ABI 7500 Real-Time PCR System (Applied Biosystem, CA, USA). Next, we performed a real-time fluorescence amplification refractory mutation system-polymerase chain reaction. Each rearrangement was further confirmed by direct Sanger sequencing; the results of *RET* rearrangement to be tested were finally read.

### 2.8 Statistical analysis

Descriptive statistics of continuous variables were expressed as mean ± standard deviation; categorical variables were presented as median (interquartile range) and frequency (%). The independent sample *t*-test was used for continuous factors with normal distribution; the Mann–Whitney *U* test was used for continuous factors without normal distribution. The categorical variables were compared using the χ^2^ test or Fisher exact test. The Delong’s test was used to compare the area under the curve (AUC). The Hosmer–Lemeshow test was used to assess whether the expected and actual probabilities were calculated with the prediction model. P < 0.05 was considered statistically significant.

## 3 Results

### 3.1 Features statistics

We enrolled 650 patients with PTC: 103 patients had *RET*/PTC rearrangement and 547 had non-*RET*/PTC rearrangement. **Table 1** shows the clinical features of all patients. Significant differences were noted in the clinical characteristics between the two cohorts, including age, tumor size, sex, TPOAb, TGAb, echogenicity, vertical and horizontal diameter ratio, calcification, and ACR-TI-RADS score.

**Table 1:** The clinical features of all enrolled patients.

Feature name	All	*RET*/PTC rearrangement		p-value
		No	Yes	
**Age, mean ± SD (years)**	42.8 ± 11.2	43.7 ± 11.0	37.9 ± 11.2	<0.001
**Tumor size, mean ± SD (mm)**	9.3 ± 6.9	8.5 ± 6.4	13.6 ± 7.9	<0.001
Sex				
** Female**	505 (77.7)	417 (76.2)	88 (85.4)	0.0396
** Male**	145 (22.3)	130 (23.8)	15 (14.6)	
TPOAb				
** Normal**	520 (80.0)	472 (86.3)	48 (46.6)	<0.001
** Abnormal**	130 (20.0)	75 (13.7)	55 (53.4)	
TGAb				
** Normal**	513 (78.9)	459 (83.9)	54 (52.4)	<0.001
** Abnormal**	137 (21.1)	88 (16.1)	49 (47.6)	
Primary site				
** Right lobe**	334 (51.4)	290 (53.0)	44 (42.7)	0.1446
** Isthmus**	22 (3.4)	14 (2.6)	8 (7.8)	
** Left lobe**	294 (45.2)	243 (44.4)	51 (49.5)	
Tumor location				
** Upper pole**	162 (24.9)	140 (25.6)	22 (21.4)	0.2072
** Lower pole**	221 (34.0)	188 (34.4)	33 (32.0)	
** Middle**	267 (41.1)	219 (40.0)	48 (46.6)	
Composition				
** Mixed cystic and solid**	23 (3.5)	19 (3.5)	4 (3.9)	0.8384
** Cystic**	1 (0.2)	1 (0.2)	0 (0)	
** Solid**	626 (96.3)	527 (96.3)	99 (96.1)	
Echogenicity				
** Hypoechoic**	610 (93.9)	526 (96.2)	84 (81.6)	<0.001
** Isoechoic**	30 (4.6)	20 (3.7)	10 (9.7)	
** Hyperechoic**	10 (1.5)	1 (0.1)	9 (8.7)	
Tumor margin				
** Irregular**	319 (49.1)	278 (50.8)	41 (39.8)	0.4145
** Ill-defined**	269 (41.4)	215 (39.3)	54 (52.4)	
** Smooth**	62 (9.5)	54 (9.9)	8 (7.8)	
The vertical and horizontal diameter ratio				
** <1**	300 (46.2)	219 (40.0)	81 (78.6)	<0.001
** ≥1**	350 (53.8)	328 (60.0)	22 (21.4)	
Shape				
** Irregular**	220 (33.9)	173 (31.6)	47 (45.6)	0.2833
** Ill-defined**	283 (43.5)	255 (46.6)	28 (27.2)	
** Regular**	147 (22.6)	119 (21.8)	28 (27.2)	
Calcification				
** Macrocalcification**	62 (9.5)	54 (9.9)	8 (7.8)	<0.001
** Microcalcification**	209 (32.2)	170 (31.1)	39 (37.9)	
** Non-calcification**	235 (36.2)	227 (41.5)	8 (7.7)	
** Cluster calcification**	144 (22.1)	96 (17.5)	48 (46.6)	
ACR-TI-RADS category				
** 3 (Mildly suspicious)**	18 (2.8)	13 (2.4)	5 (4.9)	<0.001
** 4 (Moderately suspicious)**	367 (56.4)	330 (60.3)	37 (35.9)	
** 5 (Highly suspicious)**	265 (40.8)	204 (37.3)	61 (59.2)	

PTC, papillary thyroid carcinoma; TGAb, thyroglobulin antibody; TPOAb, thyroid peroxidase antibody; ACR-TI-RADS, American College of Radiology Thyroid Imaging Reporting and Data System.

In radiomics, 1477 handcrafted features were extracted, including 306 first-order features, 14 shape features, and the last texture features. In DTL, we compared and visualized the correlation coefficients of the deep learning features. We established that the collinearity between the features was weak, indicating that deep learning still further captured the differences.

**Figure 3:**
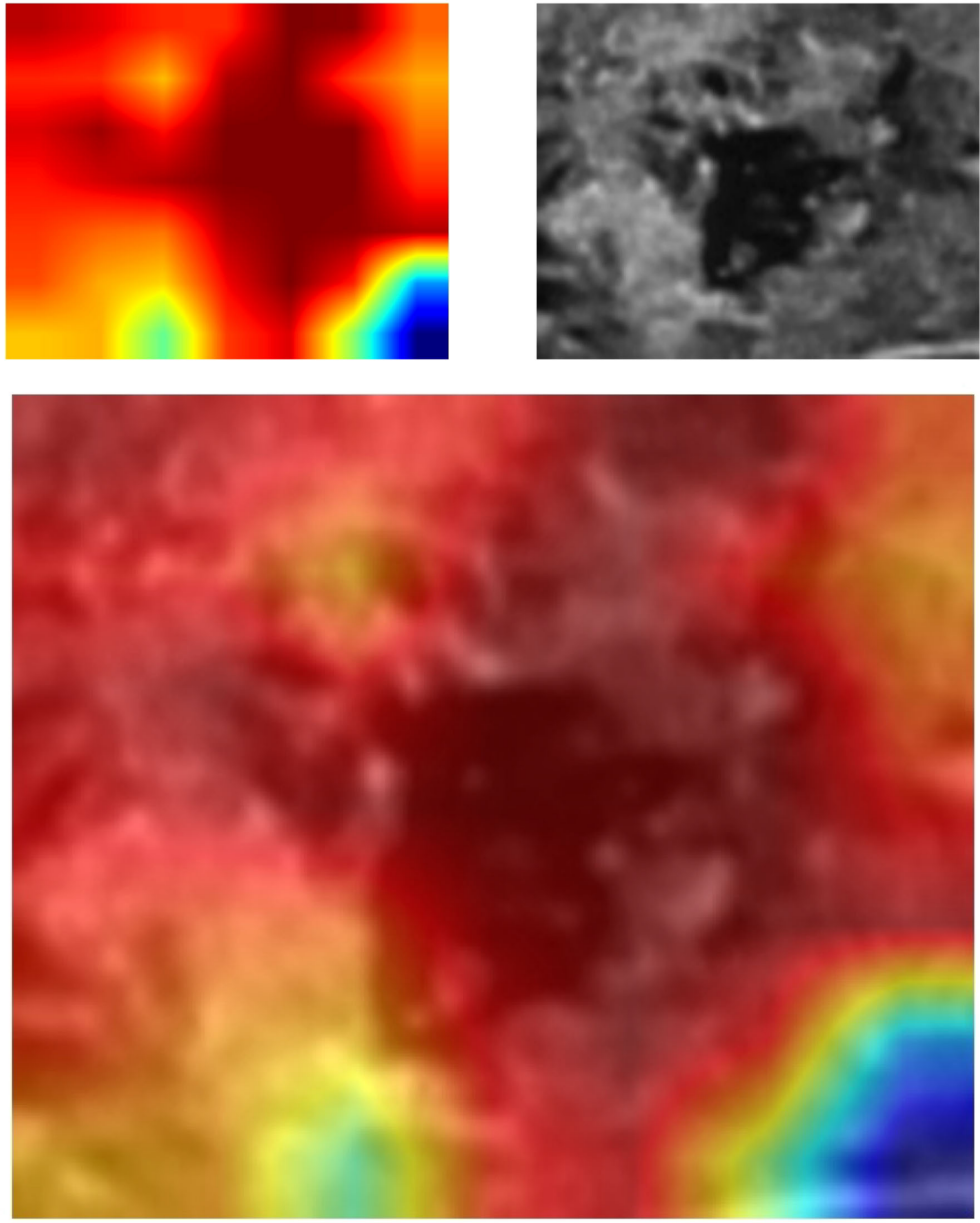
Grad-CAM visualization. Grad-CAM, gradient-weighted class activation mapping.

For investigating the interpretability of the DLR, we visualized the network by applying the gradient-weighted class activation mapping, which could provide a rough localization map highlighting the import regions for the classification target. The last convolutional layer of the last res-block was made transparent (**Figure 3**).

**Figure 4:**
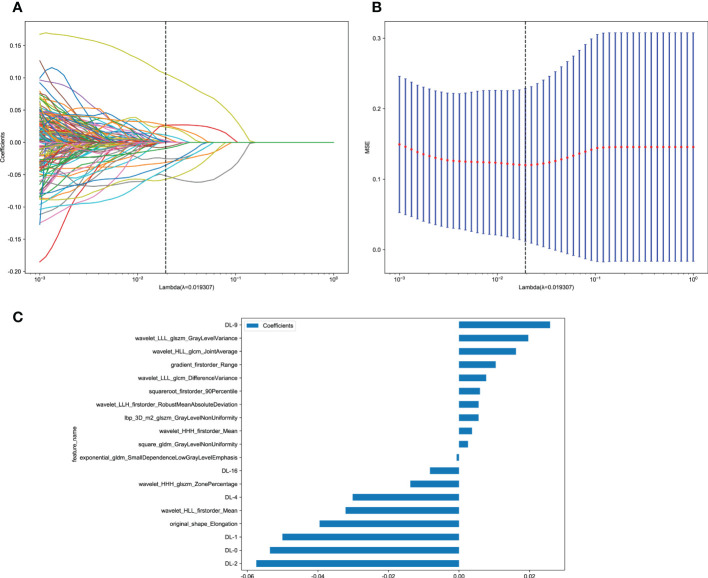
Deep learning radiomics (DLR) feature selection using the least absolute shrinkage and selection operator (LASSO) logistic regression model in the training cohort and the histogram of the DLR-score based on the selected features. **(A)** Coefficients of 10-fold cross-validation. **(B)** Mean square error of 10-fold validation. **(C)** The histogram of the DLR-score based on the selected features. Depending on the regulation weight λ, LASSO shrinks all regression coefficients towards zero and sets the coefficients of many irrelevant features exactly to zero. To find an optimal λ, 10-fold cross-validation with minimum criteria was employed, where the final value of λ yielded minimum cross-validation error. The retained features with nonzero coefficients were used for regression model fitting and combined into a DLR signature. Subsequently, we obtained a DLR score for each patient by a linear combination of retained features weighed by their model coefficients.

Next, 19 features of nonzero coefficients were selected from radiomics features and deep learning features to obtain the DLR-score with a LASSO logistic regression model in the training cohort. Coefficients, mean standard error of 10 folds validation, and the value of the coefficients in the final selected none zero features are shown in **Figure 4**. The DLR score is shown below:

DLR_score = 0.15893069804681456–− 0.000667 × exponential_gldm_SmallDependenceLowGrayLevelEmphasis + 0.010403 × gradient_firstorder_Range + 0.005532 × lbp_3D_m2_glszm_GrayLevelNonUniformity − 0.039521 × original_shape_Elongation + 0.002556 × square_gldm_GrayLevelNonUniformity + 0.005940 × squareroot_firstorder_90Percentile + 0.003671 × wavelet_HHH_firstorder_Mean − 0.013805 × wavelet_HHH_glszm_ZonePercentage − 0.032163 × wavelet_HLL_firstorder_Mean + 0.016144 × wavelet_HLL_glcm_JointAverage + 0.005555 × wavelet_LLH_firstorder_RobustMeanAbsoluteDeviation + 0.007702 × wavelet_LLL_glcm_DifferenceVariance + 0.019646 × wavelet_LLL_glszm_GrayLevelVariance − 0.053602 × DL-0 − 0.050075 × DL-1 − 0.057482 × DL-2 − 0.030163 × DL-4 + 0.025809 × DL-9 − 0.008247 × DL-16

### 3.2 Signature efficiency comparison

A 5-fold cross-validation method was used; we divided all patients into the training and test cohorts, and the test cohort was to be fixed for a fair comparison. To compare the efficiency of each signature, we further selected the best model from each signature-building process.

**Figure 5:**
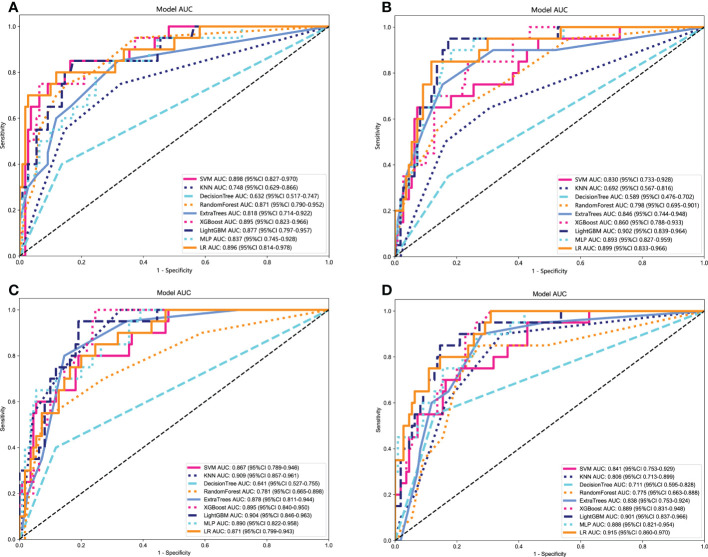
Receiver operating characteristic (ROC) curves of different models in the test cohort. **(A)** ROC curves of different models on Clinical signature. **(B)** ROC curves of different models on Rad signature. **(C)** ROC curves of different models on DTL signature. **(D)** ROC curves of different models on DLR signature.

The optimal model was obtained using radiomics features compared with an LR, SVM, KNN, Decision Tree, Random Forest, Extra Trees, XGBoost, and LightGBM classifier. The features of other categories were similarly related and modeled. LR performs almost the best performance in each model of the *RET*/PTC rearrangement respectively. **Figure 5** shows the receiver operating characteristic analysis of different models on the test cohort.

### 3.3 Establishment and validation of the Nomogram

**Figure 6:**
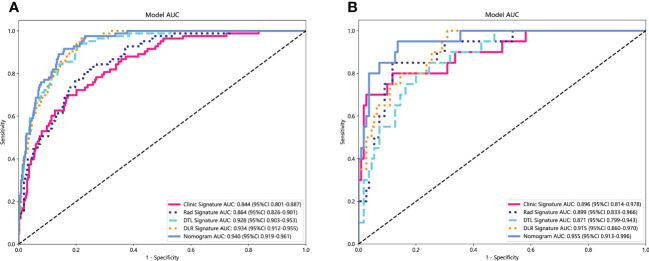
Receiver operating characteristic (ROC) curves of clinic signature, Rad signature, DTL signature, DLR signature and Nomogram. **(A)** in training cohort; **(B)** in test cohort.

In the training cohort, with both clinical signature AUC = 0.8442 [95% confidence interval (CI): 0.8009–0.8874] and radiomics signature AUC = 0.8638 (95% CI: 0.8262–0.9014), DLR is combined radiomics and DTL features achieved AUC = 0.9335 (95% CI: 0.9119–0.9551). In the test cohort, with both clinical signature AUC = 0.8959 (95% CI: 0.8141–0.9777) and radiomics signature AUC = 0.8991 (95% CI: 0.8325–0.9656), DLR was also the best model between radiomics signature and DLR signature [(DLR: 0.9150) vs. (Rad: 0.8991, DTL: 0.8709)]. DLRN using the logistic regression algorithm was performed to combine the clinical signature and DLR signature, which shows that the best performance AUC was 0.9545 (95% CI 0.9133–0.9958). **Table 2** shows all models that we used to predict the *RET*/PTC rearrangement. **Figure 6** shows the AUC in both the training and test cohorts. To compare the clinical signature, DTL signature, DLR signature, radiomics signature, and Nomogram, the Delong test was used (**Table 3**).

**Table 2:** Predictive efficacy of all models in training cohort and test cohort.

	Training Cohort					Test Cohort				
Signature	Clinic Signature	Rad Signature	DTL Signature	DLR Signature	Nomogram	Clinic Signature	Rad Signature	DTL Signature	DLR Signature	Nomogram
**Accuracy**	0.8096	0.7615	0.8192	0.8154	0.8538	0.8692	0.8769	0.7692	0.7385	0.8769
**AUC**	0.8442	0.8638	0.9283	0.9335	0.9396	0.8959	0.8991	0.8709	0.9150	0.9545
**95%CI**	0.8009 - 0.8874	0.8262 - 0.9014	0.9035 - 0.9531	0.9119 - 0.9551	0.9185 - 0.9607	0.8141 - 0.9777	0.8325 - 0.9656	0.7986 - 0.9432	0.8601 - 0.9699	0.9133 - 0.9958
**Sensitivity**	0.6988	0.8313	0.9398	0.9639	0.9157	0.8000	0.8500	0.8500	1.0000	0.9500
**Specificity**	0.8307	0.7483	0.7963	0.7872	0.8421	0.8818	0.8818	0.7545	0.6909	0.8636
**PPV**	0.4394	0.3855	0.4671	0.4624	0.5241	0.5517	0.5667	0.3864	0.3704	0.5588
**NPV**	0.9356	0.9589	0.9858	0.9914	0.9813	0.9604	0.9700	0.9651	1.0000	0.9896
**Precision**	0.4394	0.3855	0.4671	0.4624	0.5241	0.5517	0.5667	0.3864	0.3704	0.5588
**Recall**	0.6988	0.8313	0.9398	0.9639	0.9157	0.8000	0.8500	0.8500	1.0000	0.9500
**F1**	0.5395	0.5267	0.6240	0.6250	0.6667	0.6531	0.6800	0.5313	0.5405	0.7037
**Threshold**	0.2115	0.1400	0.1255	0.1103	0.1123	0.2194	0.1974	0.0798	0.0876	0.1463

AUC, area under curve; CI, confidence interval; PPV, positive predictive value; NPV, negative predictive value.

**Table 3:** Delong test for each model.

Cohort	Nomogram Vs Clinical	Nomogram Vs Rad	Nomogram Vs DTL	Nomogram Vs DLR
**Train**	<0.0001	<0.0001	0.2898	0.2027
**Test**	0.1020	0.0771	0.0176	0.0626

**Figure 7:**
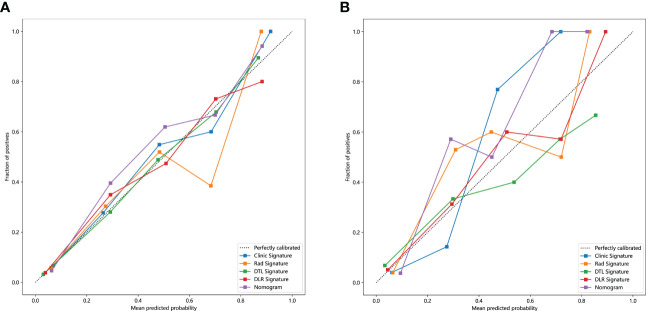
The calibration curves clinic signature, Rad signature, DTL signature, DLR signature and Nomogram. **(A)** in the training cohort; **(B)** in the test cohort.

The Nomogram calibration curves showed good agreement between the predicted and observed *RET*/PTC rearrangement in the training and test cohorts. The *p*-values of the Hosmer–Lemeshow test were 0.5655, 0.4756, 0.3451, 0.9988, and 0.2142 inspections of clinical signature, radiomics signature, DTL signature, DLR signature, and Nomogram (**Table 4**). This shows that Nomogram perfectly fits in both the training and test cohorts. **Figure 7** shows the calibration curves in the training and test cohorts.

**Table 4:** Hosmer–Lemeshow test.

Cohort	Clinic Signature	Rad Signature	DTL Signature	DLR Signature	Nomogram
**Train**	0.8457	0.7195	0.9141	0.5513	0.0787
**Test**	0.5655	0.4756	0.3451	0.9988	0.2142

**Figure 8:**
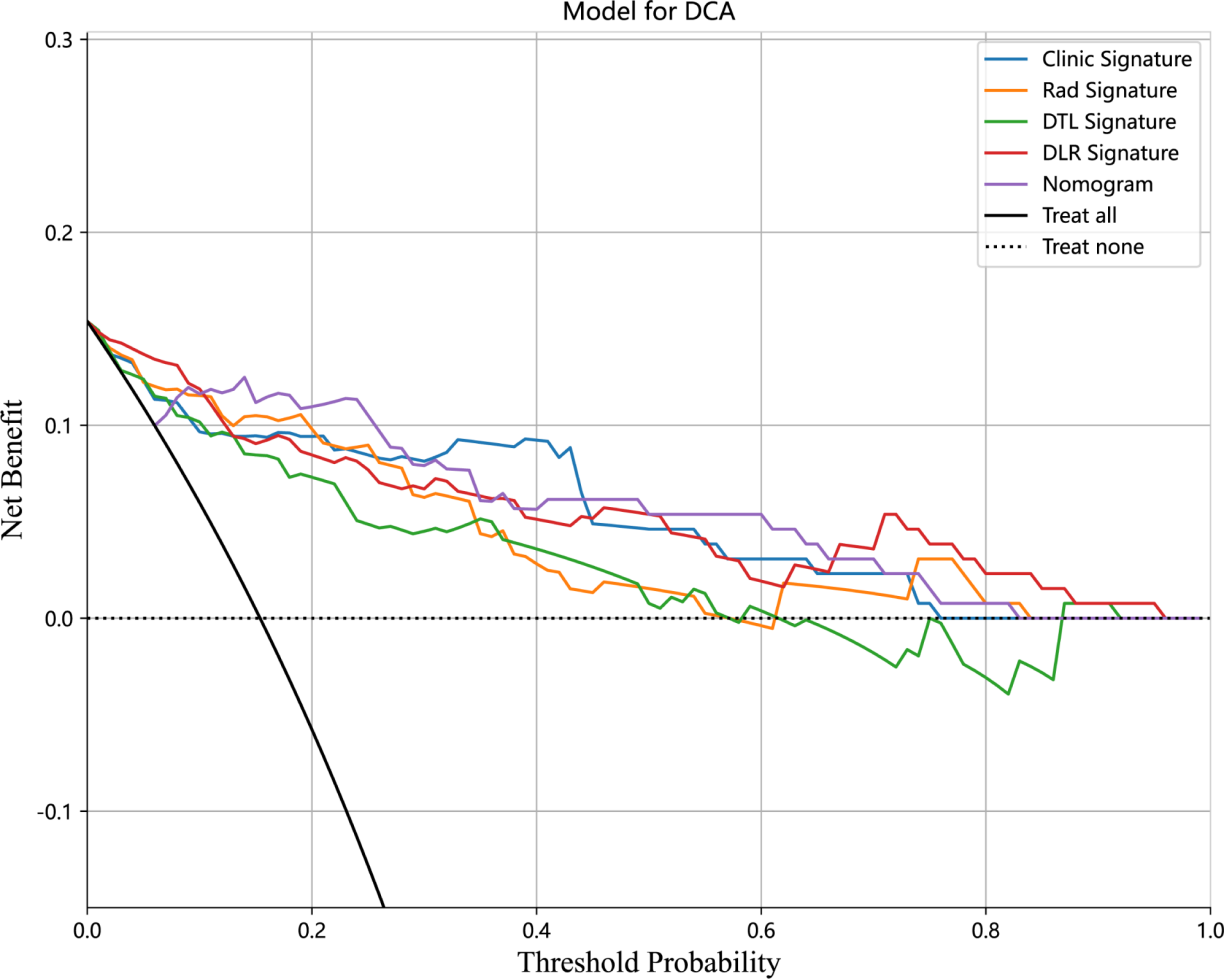
Decision curve analysis (DCA) for the Clinical signature, Rad signature, DTL signature, DLR signature, and Nomogram in the test cohort.

We also evaluated each model through DCA. DCA for the clinical signature, radiomics signature, DTL signature, DLR signature, and Nomogram is presented in **Figure 8**. The preoperative prediction of *RET*/PTC rearrangement using a radiomics nomogram has been shown to have better clinical benefits.

**Figure 9:**
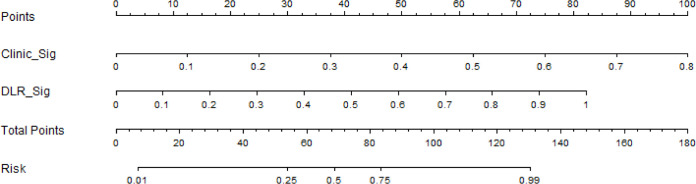
The nomogram to predict *RET*/PTC rearrangement.

Based on the clinical signature and DLR signature, we established the nomogram to predict the *RET*/PTC rearrangement (**Figure 9**).

## 4 Discussion

Most patients with PTC have an excellent prognosis for long-term survival; however, some subtypes of PTC have an aggressive tumor process wherein *RET* rearrangements are positively associated with high-risk pathological factors for PTC, such as early occurrence, large tumor, rapid growth, and high metastatic capacity (12). *RET* rearrangements play an important role in the occurrence and development of PTC and can be used as a significant indicator for the diagnosis of PTC. The *RET*/PTC3 rearrangement is more aggressive than the *RET*/PTC1 rearrangement, among the most common subtypes of *RET* rearrangements (14). Therefore, preoperative determination of *RET* rearrangement will successfully assist in making more aggressive treatment strategies for patients with high-risk PTC. However, due to the low incidence of *RET* rearrangement in sporadic PTC (approximately 20%), as a routine preoperative examination, the *RET* gene test is not of good clinical utility.

In this study, we created a novel model built by ultrasound radiomics combined with DTL for the preoperative prediction of *RET* rearrangement in patients with PTC. We developed and validated five models, the clinical, radiomics, DTL, DLR, and DLRN signatures, for the prediction of the presence of *RET*/PTC rearrangement by quantitative analysis of thyroid ultrasound images. In both training and test cohorts, DLRN demonstrated the best-predicted performance compared with the other models. The AUC of DLRN in the test cohort could reach 0.9545 (95% CI: 0.9133–0.9558) in the test cohort and 0.9396 (95% CI: 0.9185–0.9607) in the training cohort. DCA showed that DLRN can improve preoperative *RET* rearrangement prediction. Thus, our results are valuable and can be distinguished from previous studies as the first attempt at combining DLR based on ultrasound images and the clinically rare *RET*/PTC rearrangement; we also demonstrated the clinical feasibility of DLRN. Furthermore, our study provides a preoperative method to non-invasively assess *RET* information and assist in design-making when clinicians are faced with ultrasound images that are difficult to determine with the naked eye.

Radiomics uses high-throughput automated extraction algorithms to evaluate the geometry, texture, and echo intensity of nodules; it also shifts from the traditional use of images for visual interpretation to their conversion to quantitative features (25, 26). Radiomics has been used to predict the molecular characteristics of various tumors (27). Concerning thyroid, radiomics has been proposed based on ultrasound images to predict the *BRAF* mutation (19, 20, 28); however, the models showed limited ability. Moreover, the *BRAF* mutation is the most representative mutation in PTC and has low specificity among all molecular features. Meanwhile, the significance of traditional ultrasound features for prediction is neglected. Although conventional ultrasound examinations rely only on the radiologist’s visual description of the nodal features and cannot dig deeper into the information and the interpretation of ultrasound images is operator-dependent, there is interobserver variability. By designing the DLR model using ultrasound images and clinical factors, we simultaneously incorporated the DLR and traditional ultrasound features. In this study, four traditional ultrasound features, such as hypoechoic, vertical and horizontal diameter ratio of <1, cluster calcification, and ACR-TI-RADS 5 (highly suspicious), were associated with *RET*/PTC rearrangement. However, data on the association between *RET* rearrangement and traditional ultrasound features of PTC are very scarce and frequently inconsistent (16). Previous studies have pointed out that the *BRAF* mutation of PTC is associated with ultrasound features, such as hypoechoic, microcalcification, and irregular margins (29). Therefore, the traditional ultrasound features with *RET*/PTC rearrangement in our study were not very representative; however, our results can be used as a reference for further study of *RET*/PTC rearrangement in ultrasound radiomics. Compared with the DLR signature based on only containing DLR features, incorporating traditional ultrasound features of the nomogram showed a better predictive performance. The ultrasound features are supposed to be included in the analysis along with radiomics parameters for enhancing the diagnostic ability of gene mutation.

Deep learning has shown remarkable progress in medical image analysis, advancing the field forward at a quick pace. DLR has more advantages than hand-crafted and radiomics features. For example, deep learning can extract multilevel features from original images *via* a hierarchical neural network and automatically identify tumor boundaries. In this study, 19 features with nonzero coefficients were filtered from radiomics and DTL features to create the DLR signature. Among the selected DTL features, there were four most significant and robust features associated with *RET* rearrangement. For the selected radiomics features, most reflected the image texture and voxel intensity. The human visual system cannot recognize these features; however, these DLR features can serve as an auxiliary tool for the prediction of *RET* rearrangement in PTC.

There are certain limitations to our study: (1) First, the samples of *RET* rearrangement were not compared by different subtypes, such as *RET*/PTC1 and *RET*/PTC3, due to the insufficient sample size, concluding that this study could only perform primary screening of patients with *RET* rearrangement in PTC and not predict the specific subtypes. (2) Second, as this was a retrospective study, a selection bias may exist. Therefore, we aim to conduct a prospective study in the future to control for confounding variables. (3) Lastly, there was a lack of external validation as it was a single-center, small-sample study; therefore, this model needs to be further validated in a multi-center, larger sample size survey.

## Data availability statement

The raw data supporting the conclusions of this article will be made available by the authors, without undue reservation.

## Ethics statement

The studies involving human participants were reviewed and approved by The Ethics Committee of the First Affiliated Hospital of Zhengzhou University. Written informed consent from the participants’ legal guardian/next of kin was not required to participate in this study in accordance with the national legislation and the institutional requirements.

## Author contributions

JY: collected the all data from our hospital, statistical analysis, and drafting of the manuscript. YZ: contributed to analysis and manuscript preparation. JZ and MJ: the accuracy of the data and proof reader. XL: supervision and proof reader. All authors contributed to the article and approved the submitted version.
