# Supplementary material for: Ultrasound images-based deep learning radiomics nomogram for preoperative prediction of RET rearrangement in papillary thyroid carcinoma
Source: Front Endocrinol (Lausanne). 2022 Dec 20;13:1062571. doi: 10.3389/fendo.2022.1062571 (PMC9807879; doi:10.3389/fendo.2022.1062571)
Supplement: Supplementary file 1 [file DataSheet_1.docx]

Supplementary Material

# Supplementary Data

**Cosine decay learning rate algorithm**

Because of the leak of image data, to better carry out the generalization, we carefully set the learning rate. We adapt the cosine decay learning rate algorithm in this study. Our learning rate is presented as follows:

$$\eta_{t}^{task-spec}=\eta_{min}^{i}+\frac{1}{2}(\eta_{max}^{i}-\eta_{min}^{i})(1+cos(\frac{T_{cur}}{T_{i}}\pi))$$

$\eta_{min}^{i}=0$, $\eta_{max}^{i}$ =0.01, $T_{i}=30$ Represents the minimum learning rate, the maximum learning rate, and the number of iteration epochs, respectively. Because the backbone part adopts pre-training parameters, to ensure the migration effect. On $T_{cur}=\frac{1}{2}T_{i}$Fine tune the parameters of the backbone part. Therefore, the learning rate of the backbone part is as follows:

$$\eta_{t}^{backbone}=\left\{ \begin{matrix} 0 & \text{ if }T_{cur}\leq\frac{1}{2}T_{i} \\ \eta_{min}^{i}+\frac{1}{2}(\eta_{max}^{i}-\eta_{min}^{i})(1+cos(\frac{T_{cur}}{T_{i}}\pi)) & \text{ if }T_{cur}>\frac{1}{2}T_{i} \end{matrix} \right.$$

Other hyperparameter configurations are as follows: optimizer: SGD, loss function: sigmoid cross entropy.

# Supplementary Tables

**Supplementary Table 1.** The supplement presents the detailed process of calculating the ACR-TI-RADS score

| Score | Compositing | Echogenicity | Shape | Margin | Echogenic Foci |
| --- | --- | --- | --- | --- | --- |
| 0 | Cystic or almost completely cystic; Spongiform | Anechoic | Wilder-than-tall | Smooth | None or large comet-tail artifacts |
| 1 | Mixed cystic and solid | Hyperechoic or isoechoic | | Ill-defined | Macrocalcifications |
| 2 | Solid or almost completely solid | Hypoechoic |  | Lobulated or irregular | Peripheral(rim) calcifications |
| 3 |  | Very hypoechoic | Taller-than-wide | Extra-thyroidal extension | Punctate echogenic foci |

ACR-TI-RADS: American College of Radiology Thyroid Imaging Reporting and Data System

**Supplementary Table 2. ACR-TI-RADS risk stratification system and management recommendations**

| Category | TI-RADS score | Follow size cutoff | FNA size cutoff |
| --- | --- | --- | --- |
| Highly suspicious | 7 points or more | 0.5 cm | 1 cm |
| Moderately suspicious | 4 to 6 points | 1 cm | 1.5 cm |
| Mildly suspicious | 3 points | 1.5 cm | 2.5 cm |
| Not suspicious | 2 points | Not indicated | Not indicated |
| Benign | 0 points | Not indicated | Not indicated |

# Supplementary Figures

**Supplementary Figure 1.** Spearman correlation coefficients of each clinical feature demonstrated no obvious linear relationship between these clinical features.


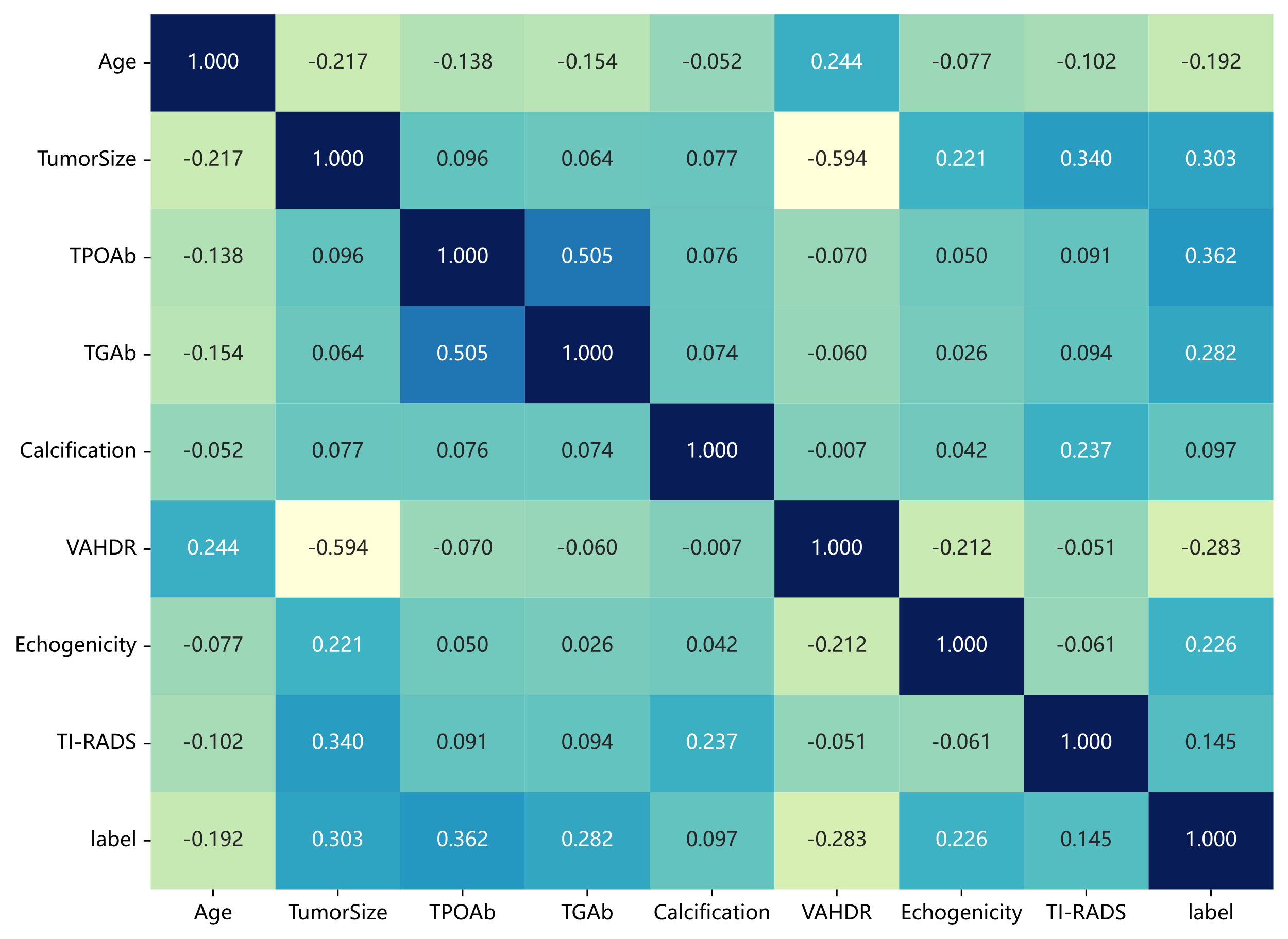


VAHDR: Vertical and horizontal diameter ratio

**Supplementary Figure 2.** Hand-crafted feature


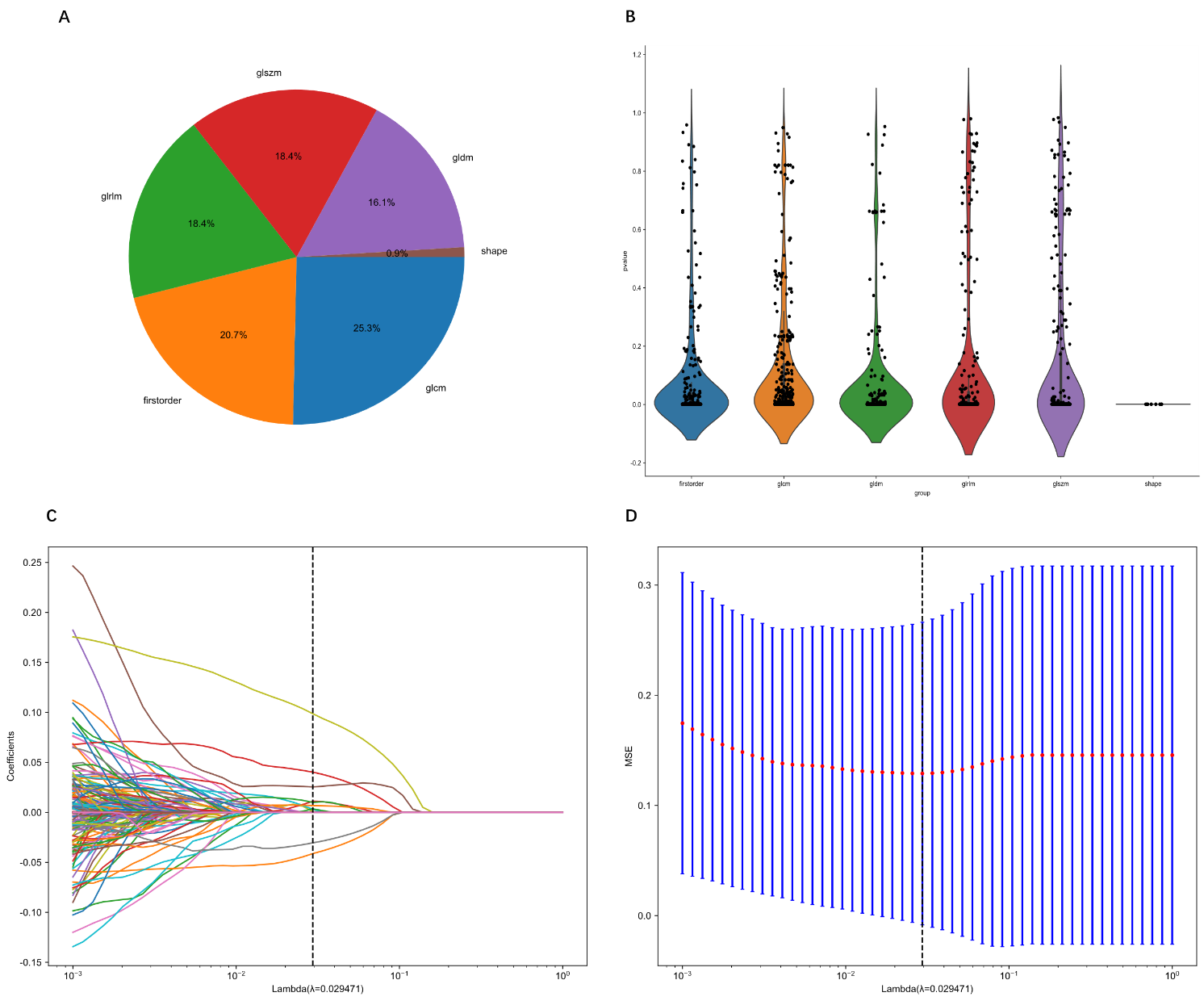


(A) Number and the ratio of handcrafted features. (B) Statistics of radiomics features. (C) Coefficients of 10-fold cross-validation. (D) MSE of 10-fold validation.

**Supplementary Figure 3.** The correlation coefficient of each deep transfer learning features
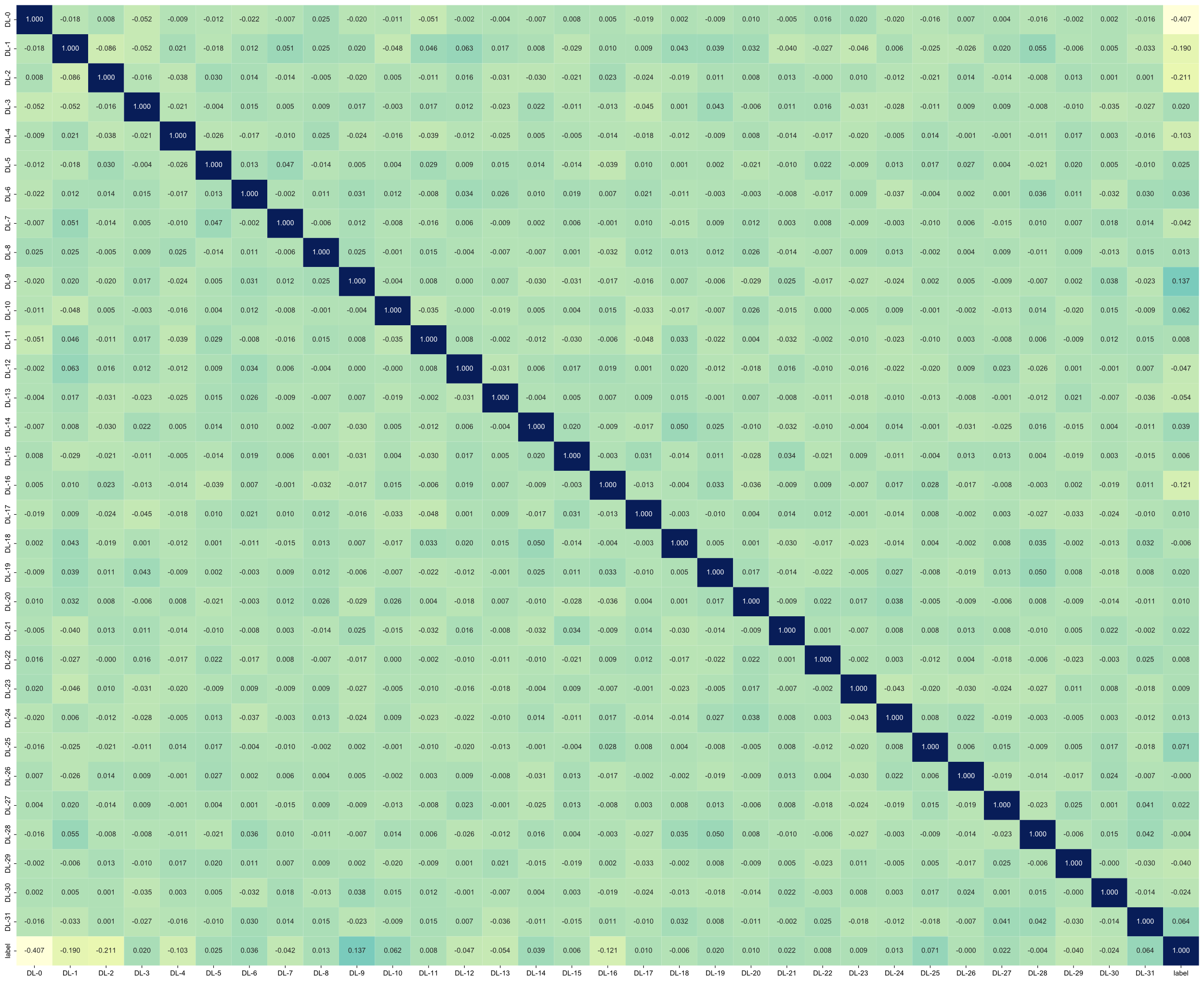


**Correlation**: For features with high repeatability, Spearman's rank correlation coefficient was also used to calculate the correlation between features, and one of the features with a correlation coefficient greater than 0.9 between any two features is retained. To retain the ability to depict features to the greatest extent, we use a greedy recursive deletion strategy for feature filtering, that is, the feature with the greatest redundancy in the current set is deleted each time.
